# A Novel Inulin-Mediated Ethanol Precipitation Method for Separating Endo-Inulinase From Inulinases for Inulooligosaccharides Production From Inulin

**DOI:** 10.3389/fbioe.2021.679720

**Published:** 2021-04-29

**Authors:** Xin Li, Qiannan Zhang, Wei Wang, Shang-Tian Yang

**Affiliations:** ^1^Jiangsu Co-innovation Center of Efficient Processing and Utilization of Forest Resources, College of Chemical Engineering, Nanjing Forestry University, Nanjing, China; ^2^Jiangsu Province Key Laboratory of Green Biomass-Based Fuels and Chemicals, Nanjing, China; ^3^William G. Lowrie Department of Chemical and Biomolecular Engineering, The Ohio State University, Columbus, OH, United States

**Keywords:** ethanol gradient precipitation, inulin, inulinase, inulooligosaccharides, biomass

## Abstract

Inulin is a kind of polysaccharide that can be obtained various biomass. Inulooligosaccharides (IOS), a kind of oligosaccharides that can be obtained from inulin by enzymatic hydrolysis using inulinases, have been regarded as the functional food ingredients. Commercially available inulinases produced by natural *Aspergillus niger* contained both endo- and exo-inulinase activities. For IOS production from inulin, it is desirable to use only endo-inulinase as exo-inulinase would produce mainly the monosacchairde fructose from inulin. In the present study, a simple inulin-mediated ethanol precipitation method was developed to separate endo- and exo-inulinases present in natural inulinases. IOS production from inulin using the enriched endo-inulinase was then optimized in process conditions including pH and temperature, achieving a high yield of ∼94%. The resultant IOS products had a degree of polymerization ranging from 2 to 7. The study demonstrated a novel method for obtaining partially purified or enriched endo-inulinase for IOS production from inulin in an efficient process.

## Introduction

Inulins are polysaccharides derived from biomass, where they function as energy storage within plant biology. The chemical structures of inulin involves a mixture of linear polymers composed of fructose residues which are linked by β-2,1-glycosidic bonds, with a glucose residue through a sucrose-type linkage at the reducing end (Chi et al., 2011). Inulin can be used directly as functional ingredients for food, meaning their inclusion into edible materials imparts a benefit to human health upon consumption, such as function as dietary fiber, relieving constipation, stimulating the growth of *Bifidobacteria* and *Lactobacillus* sp. in the colon, etc (Shoaib et al., 2016; Singh et al., 2016). It is also possible to convert inulin into valuable biologically derived chemicals, such as inulooligosaccharides (IOS), citric acid, butanediol, L-lactic acid, D-lactic acid, and more (Chi et al., 2011; Zheng et al., 2018; Singh et al., 2019).

In the recent years, IOS in particular has gained more attention for their value in functional foods. This increased interest is attributable to IOS’ bifidogenic nature and health-benefiting properties. Inulin and oligofructose are “Generally Regarded As Safe (GRAS)”, and used as either supplements or macronutrient substitutes (Coussement, 1999). Taking into account IOS’ cost, sustainable feedstock and abundance in natural sources, inulin is considered to be a promising substrate for production of IOS (Singh et al., 2016). Inulin can be enzymatically converted to IOS using a controlled hydrolysis process involving endo-inulinase, a conversion that is known to be a single step process (Singh et al., 2016). This means of conversion can be seen as having advantages when considering it from an industrial point of view, primarily due to the reaction conditions and kinetic simplicity of conversion. However, it is most certainly important to highlight the key cost driver in scaling up this conversion process: the preparation of endo-inulinase. natural inulinase is generally composed of endo-inulinase and exo-inulinase activity, as reported in a recent study (Flores et al., 2016). Inulinase-driven hydrolysis of inulin is achieved by the mechanisms of both exo-inulinase (EC 3.2.1.80), which cleaves fructose from the non-reducing end of inulin, and endo-inulinase (EC 3.2.1.7), which breaks the internal linkage of inulin to release intermediate short-chain IOS (Mutanda et al., 2008). Consequently, fructose is the major product with a little IOS in the hydrolyzate after hydrolysis of inulin by natural inulinase.

To obtain high yields of IOS from inulin, an ideal inulinase should lack extracellular exo-inulinase activity to promote more endo-inulinase activity (Singh et al., 2016). Therefore, several approaches have been carried out in recent years to promote endo-inulinase functionality. One reported approach involved chromatographic isolation of endo-inulinase from natural inulinases produced by varying microorganisms (Park et al., 1999; Cho and Yun, 2002; Jin et al., 2005; Naidoo et al., 2015). Unfortunately, the use of column chromatography is an expensive and complex approach which places significant cost burden upon an IOS process (Golunski et al., 2011). A different approach included use of a recombinant endo-inulinase to produce IOS from inulin (He et al., 2014; Xu et al., 2016; Bao et al., 2019; Jiang et al., 2019). Importantly, *Aspergillus niger* and the carbohydrases it produces are recognized as GRAS by the United States Food and Drug Administration (Schuster et al., 2002). However, use of recombinant enzymes is still a subject of practical and ethical debate. This mostly eliminates the aforementioned approach from being economically viable in the short term. Therefore, it remains challenging to obtain endo-inulinase with free exo-inulinase activity without amassing a wealth of additional complications.

In the present study we underwent development of a novel (as well as simple) method to separate endo-inulinase and exo-inulinase activity from natural inulinases derived from *A. niger* (recall that it is GRAS). Our method is based on the differences in how endo-inulinase and exo-inulinase bind linear inulin chains. Endo-inulinase and exo-inulinase bind inulin molecules of different lengths, and also tend to precipitate at different ethanol concentrations. These intrinsic characteristics were manipulated to isolate endo-inulinase exclusively for production of IOS. Furthermore, the conditions of IOS production by the enriched endo-inulinase were optimized in temperature, inulin concentration, enzyme loading and reaction time. The goal of this work was to demonstrate practical means of using inulinase to produce valuable IOS from sustainably sourced inulin.

## Materials and Methods

### Materials

Natural inulinase from *A. niger* (I6285) was purchased from Sigma-Aldrich (St. Louis, MO, United States). The enzyme solution, once received, was diluted to a final inulinase activity of 12.5 U/mL and stored at 5°C prior to further experimentation. Inulin was purchased from Beneo Orafti (Tienen, Belgium). Ethanol of analytical purity was purchased from Nanjing Chemical Reagent Co., Ltd. and was used as received without further purification.

### Inulin-Mediated Ethanol Gradient Precipitation

Phase I: The initial ethanol solution (40–60%, v/v) was cooled in a water-ice bath for 30 min, and then pH adjusted to 4.6. Next, 20 mL of the chilled and pH-adjusted ethanol solution was mixed with 0.8 g inulin (Beneo Orafti, Belgium) and the natural inulinase. This mixture was kept at 0°C for 2–4 h. After time, the precipitates (named P40, P45, P50, P55, and P60, respectively) was recovered by centrifugation at 2,000–3,000 g and 0°C for 10 min, which is according to the work of Gu et al. (2020).

Phase II: Following Phase I, the supernatant (ethanol 60%, v/v) was as the initial solution of Phase II. Additional chilled ethanol (at a temperate of −20°C) was dropwise added into the supernatant to reach an ethanol concentration of 65% (v/v). This new mixture was also then kept at 0°C for 2–4 h. A newly precipitated fraction was recovered by centrifugation (same parameters as before) and dubbed P65. This process was repeated in a step-wise fashion to obtain precipitates P70, P75, and P80.

Each recovered precipitate was re-dissolved in sodium acetate buffer (pH 4.6, 100 mM) prior to determine enzyme activity or use in bioconversion. The ethanol gradient precipitation of inulin without the natural inulinase was the same process as above. All experiments were carried out in triplicate.

### Determination of Enzyme Activity

Enzyme activity was measured by proxy using the concentration of reducing sugars released from inulin and sucrose. Inulin (Beneo Orafti, Belgium) was used as the substrate for determination of inulinase activity. Sucrose (Sinopharm, China) was used as substrate for determination of sucrase activity. A reaction mixture containing 50 μL of diluted crude enzyme and 450 μL of 5% (w/v) substrate solution (dissolved in 0.1 M sodium acetate buffer, pH 4.6) was incubated at 60°C for 10 min. A denatured enzyme in inulin or sucrose solution was also used to serve as an experimental control. The amount of reducing sugars liberated from inulin or sucrose was determined using a 3,5-dinitrosalicylic acid (DNS) assay (Xu et al., 2016), with fructose serving as a standard. Assay response was measured as absorbance at 520 nm. Regarding enzyme activity derived from said assay, one unit of inulinase activity or sucrase activity was defined as the amount of enzyme required that produced 1 μmol of reducing sugar per minute under the assay conditions used in this study.

### Inulooligosaccharides Production by Enzyme Hydrolysis of Inulin

Inulin (Beneo Orafti, Belgium) was used as the substrate for IOS production. Enzymatic hydrolysis of inulin was carried out under different conditions, including temperature, substrate concentration, enzyme loading, and reaction time. Samples of the hydrolyzates (1 mL) were taken at different intervals and boiled at 100°C for 5 min to denature the enzymes and cease further bioconversion. Following denaturing, all samples were centrifuged at 10,000–12,000 *g* for 5 min, and the supernatants were analyzed by high-performance anion exchange chromatography quantitatively coupled with pulsed ampere detection (HPAEC-PAD). The details of this chromatography method are described in the following section.

### Analytical Methods

Quantitative analysis of IOS was carried out in accordance with the reported analytical method (Xu et al., 2016), albeit with some modifications. Measurement of resultant IOS concentrations was performed using a HPAEC-PAD with a CarboPac PA200 column (250 mm × 3 mm, Dionex, Sunnyvale, CA, United States). Water, 200 mM NaOH and 500 mM sodium acetate (NaAc) were used as the mobile phase. A gradient elution strategy was implemented as follows: 60% water, 40% 200 mM NaOH and 0 → 12% 500 mM NaAc in 0–5 min; 48% water, 40% 200 mM NaOH and 12% 500 mM NaAc in 5–25 min; 48 → 20% water, 40% 200 mM NaOH and 12 → 40% 500 mM NaAc in 25–30 min; 20 → 0% water, 40 → 100% 200 mM NaOH and 40 → 0% 500 mM NaAc in 30–33 min; 100% 200 mM NaOH in 33–35 min; 0 → 60% water and 100 → 40% 200 mM NaOH in 35–50 min. The column temperature was 30°C, and the flow rate was 0.4 mL/min. The method showed that IOS quantitation had a good linear relationship within the 0.1–10 mg/L concentration range. IOS yields were calculated as the ratio of total IOS (g) to the inulin (g). IOS are a group of oligosaccharides, which is formed by β-(2–1) linked fructofuranosyl unit on the end of sucrose molecule, with degree of polymerization (DP) ranging from 2 to 10 (Singh et al., 2016). In the name of simplicity, we arbitrarily defined IOS with chain lengths between 2 and 10 residues. Glucose (G), fructose (F), sucrose (GF), 1-kestose (GF2), 1-nystose (GF3), 1-F-1-β-D-fructofuranosyl nystose (GF4), 1-F-(1-β-D-fructofuranosyl) -2-nystose (GF5) and 1-F-(1-β-D-fructofuranosyl) -3-nystose (GF6) were quantitatively determined by an external standard method, and others were estimated by the ratio of their peak areas to total peak areas of HPAEC.

Gel permeation chromatography (GPC) analysis of inulin was carried out at 35°C with an Agilent 1,260 HPLC equipped with two columns (Ultrahydrogel-120 and Ultrahydrogel-250). 50 mM potassium dihydrogen phosphate was used as the mobile phase. Mobile phase flow rate was 0.6 mL/min. The DP value was calculated according to the equation as follows:

Averagemolecularweight(g/mol)=162×(DP-1)+180

### Statistical Analysis

Statistical analysis was carried out using Origin 2016 software (OriginLab Corporation, Northampton, MA 01060, United States). The data were assessed using a one-way ANOVA with Tukey’s test.

## Results and Discussion

### The Inulin-Mediated Ethanol Gradient Precipitation of Natural Inulinase From *Aspergillus niger*

*Aspergillus niger* is known to produce inulinases with high levels of endo- and exo-inulinase activities (Singh and Gill, 2006). Exo-inulinase removes one fructose from the non-reducing end of inulin, whereas endo-inulinase randomly cleaves the β-2,1-glycosidic bonds in inulin (Qiu et al., 2018). Two different hydrolytic behaviors implied that there are different binding properties existing between inulinases and inulin. To check the idea, ethanol gradient precipitation was applied to explore inulin-binding properties of inulinases. We qualitatively precipitated inulinase by ethanol gradient precipitation both in the presence and absence of inulin at ethanol concentration ranging from 40% (v/v) to 80% (v/v) (Supplementary Figure S1). An interesting phenomenon was found that precipitates were obtained at different ethanol concentrations in the presence of inulin while no precipitates were observed when the system had no inulin dissolved within. Comparably, precipitates, showing higher relative inulinase activity (in redissolved form) were also observed at ethanol concentrations of 50% (v/v) and 70% (v/v), respectively. These results demonstrate that inulinases will not precipitate if not in the presence of inulin. This suggests that some sort of association occurs between the enzymes of interest and inulin, which then allows the coordinated enzymes to be precipitated along with the substrate inulin. In addition, results in Table 1 demonstrate that longer chains of inulin are prone to precipitate at lower ethanol concentrations compared to inulin molecules of shorter length (Xu et al., 2014). Based on all of these findings, we inferred that the two precipitates might be two different inulinases, and one tend to bind the longer chain inulin molecules while the other was likely to bind the shorter chain inulin molecules. Furthermore, ethanol gradient precipitation might be an effective method to separate endo-inulinase from exo-inulinase present in the natural inulinases.

**TABLE 1:** Estimated molecular weights of inulin precipitates.

	Average molecular weight (g/mol)	Degree of polymerization
Inulin as control	3,260	20
50% (v/v) ethanol	4,730	29
70% (v/v) ethanol	3,730	22

**FIGURE 1:**
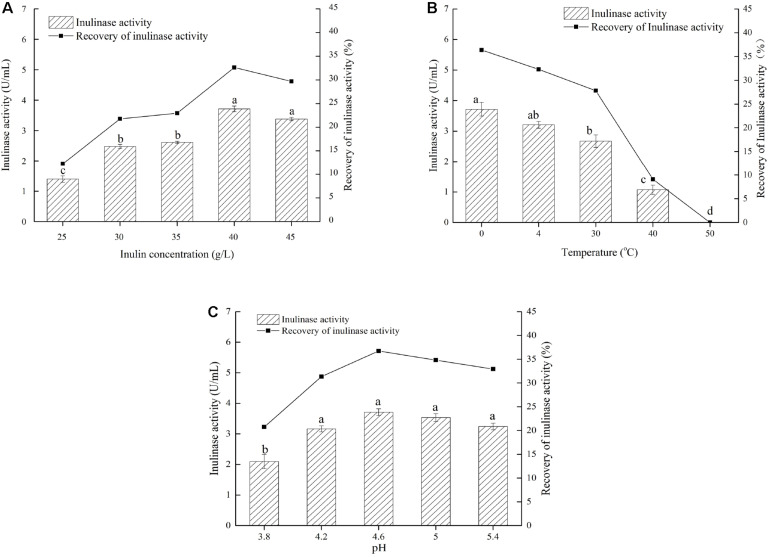
Effects of inulin concentration, temperature and pH on the inulin-mediated ethanol precipitation of natural inulinase from *Aspergillus niger.* The conditions of the inulin-mediated ethanol precipitation were as follow: **(A)** 0°C, pH 4.0–5.0 and ethanol concentration of 55% (v/v); **(B)** 40 g/L inulin, pH 4.0–5.0 and ethanol concentration of 55% (v/v); **(C)** 40 g/L inulin, 0°C and ethanol concentration of 55% (v/v). Lower case letters: Significant differences (*p* < 0.05). Statistical analysis was performed using a one-way ANOVA with Tukey’s test.

Based on the findings mentioned above, we explored a simple and quick method to separate different inulinases from natural inulinase. In the present work, inulin-mediated ethanol gradient precipitation was carried out to separate different inulinases from natural inulinase. 40 g/L inulin and 0°C were identified as the optimal conditions for inulin-ethanol precipitation (Figures 1A,B). In addition, pH 4.6 was determined to be the optimum pH for the selective isolation (Figure 1C).

**FIGURE 2:**
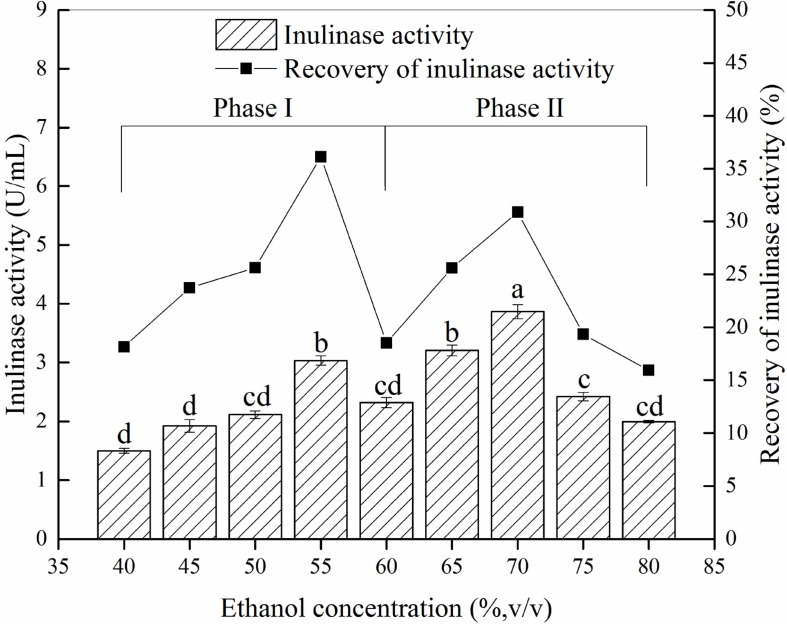
The inulin-mediated ethanol gradient precipitation of natural inulinase from *Aspergillus niger.* The conditions of the inulin-ethanol precipitation were as follows: 40 g/L inulin, 0°C and pH 4.6. Lower case letters: Significant differences (*p* < 0.05). Statistical analysis was performed using a one-way ANOVA with Tukey’s test.

Different ethanol concentration demonstrated a significantly effect on precipitation and recovery of inulinase (Figure 2). The results showed that precipitates P55 and P70 [corresponding to the material precipitated at 55% ethanol (Phase I) and 70% ethanol (Phase II), respectively] contained greater inulinase activity (Figure 2), with both recovering about 67% initial inulinase activity. However, because different proteins tend to precipitate at different aqueous ethanol concentrations (van Oss, 1989), we thus inferred that P55 (Phase I) and P70 (Phase II) could be two different inulinases.

**FIGURE 3:**
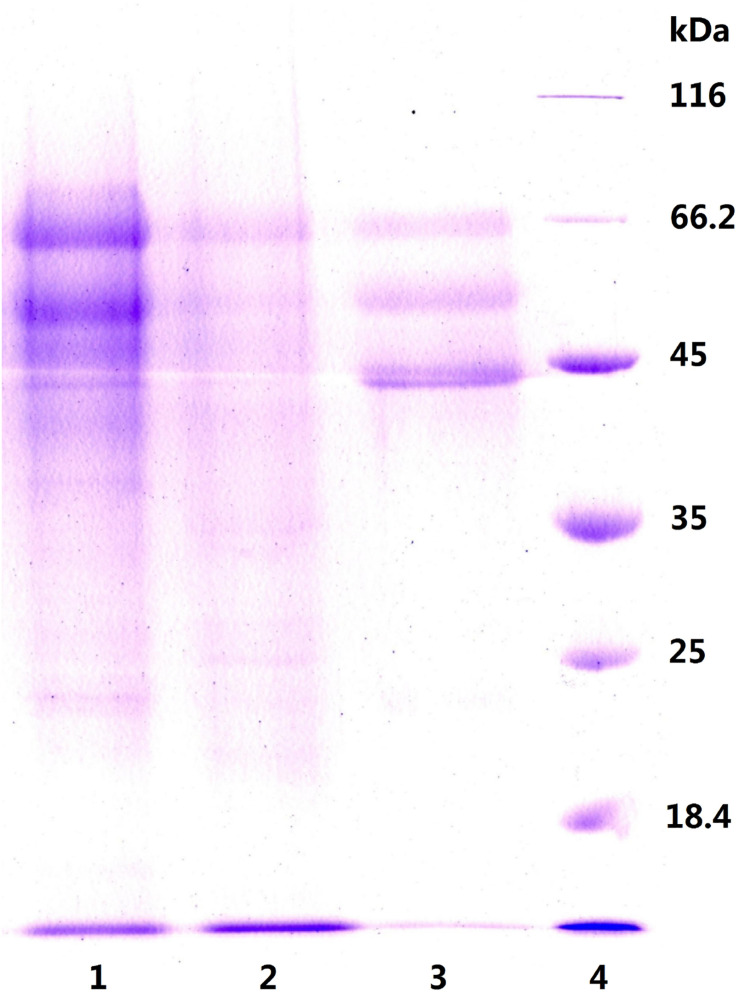
SDS-PAGE analysis of P55 and P70. Lane 1: P70, precipitate at ethanol 70% (v/v); Lane 2: P55, precipitate at ethanol 55% (v/v); Lane 3: natural inulinase of *Aspergillus niger*; Lane 4: marker.

To explore this hypothesis, we next carried out another experiment to characterize the precipitates P55 and P70 for both inulinase activity and sucrase activity (Table 2). Generally, a simple identification of endo- and exo-inulinase activity is characterized by the ratio of inulinase (I) activity: sucrase (S) activity (Ettalibi and Baratti, 1987; Kango, 2008). Ettalibi and Baratti (1987) reported that the I/S ratio of endo-inulinase was higher than that of exo-inulinase. Wang et al. (2003) also reported that the I/S ratio of exo-inulinase was lower than 10, while that of endo-inulinase was higher than 10. In our experiment, the drastic difference in I/S ratio between P70 (39) and P55 (1.9) confirmed the different enzymatic composition of P70 and P55, which was expected given that what precipitated at 70% ethanol remained in solution during P55 preparation. Nevertheless, the results suggested that P55 contained mostly exo-inulinase while P70 contained mostly endo-inulinase. According to SDS-PAGE analysis (Figure 3), P70 and P55 consisted of several different protein bands, respectively, indicating that no single protein band could be obtained by the inulin-mediated ethanol gradient precipitation.

**TABLE 2:** The different enzyme activities of precipitates from the inulin-ethanol precipitation of natural inulinase from *Aspergillus niger*.

Precipitate	Inulinase activity (I, U/mL)	Sucrase activity (S, U/mL)	I/S ratio
P55	3.0	1.6	1.9
P70	3.9	0.1	39

### Inulin Hydrolysis by Fractionated Inulinases

**FIGURE 4:**
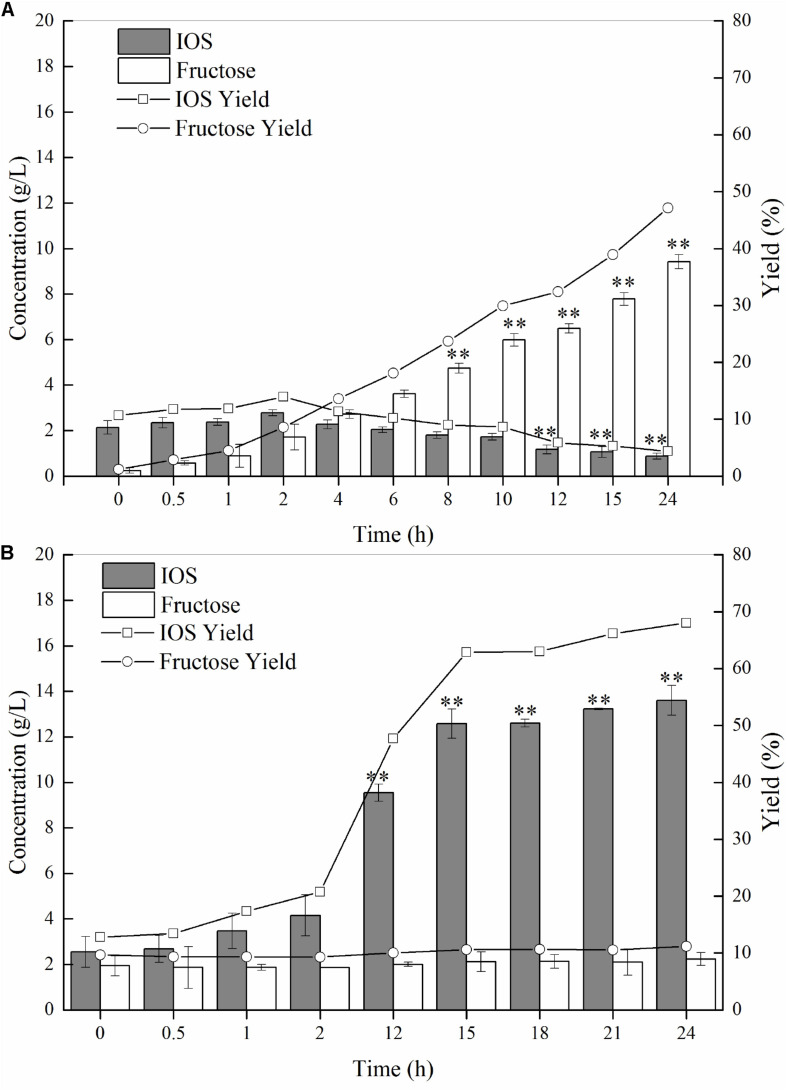
Inulin hydrolysis by P55 **(A)** and P70 **(B)**. The conditions of 24 h-enzymatic hydrolysis were as follow: 20 g/L inulin, 10 U inulinase activity per gram of inulin, 50°C and pH 4.6. Statistical analysis was performed using a one-way ANOVA with Tukey’s test to determine a significant increase or decrease of IOS or fructose compared with those at 2 h (*p* < 0.01). ^∗∗^Indicates a statistical significance.

As discussed earlier, we conjectured that P55 would exhibit strong exo-inulinase activity while P70 should demonstrate high levels of endo-inulinase activity. We tested the hydrolytic behaviors of P55 and P70 by performing enzymatic hydrolysis of inulin at the enzyme loading of 10 U/(g inulin) (Figure 4). At 0 h, it can be seen that minute quantities of IOS and fructose were already present in the inulin substrates. As shown in Figure 4A, fructose concentrations significantly increased over time while IOS concentrations gradually decreased. The behavior of P70 was completely different from that of P50, where it can be seen that IOS concentrations continued to increase with hydrolysis times while fructose concentrations remained relatively constant (Figure 4B). In bioconversion using P70, the maximum IOS concentrations and yields were obtained at 15 h (No significant difference between 15 and 24 h), and likely would have further increased given more hydrolysis time (albeit to a likely minor extent). From these observations, it can be seen that inulin was predominantly hydrolyzed into fructose by P55, whereas P70 exclusively acted upon hydrolyzing inulin to IOS. According to their respective inulin hydrolytic mechanisms, endo-inulinases act by producing IOS while exo-inulinases hydrolyze inulin and IOS to form fructose (Chi et al., 2009). Therefore, our results show that the precipitate P55 was dominated by exo-inulinase activity, and the precipitate P70 was dominated by endo-type inulinase activity. These findings further indicate that the inulin-mediated ethanol gradient precipitation method was effective for obtaining endo-inulinase activity which would be effective in producing IOS at industrial scales.

### Optimization of IOS Production by Fractionated Endo-Inulinase

**FIGURE 5:**
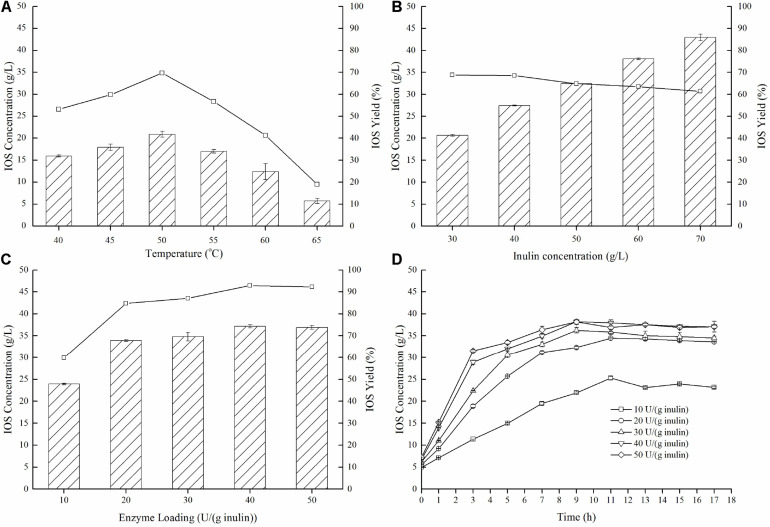
Effects of temperature **(A)**, inulin concentration **(B)**, and enzyme loading **(C,D)** on the production of IOS from inulin by the isolated endo-inulinase. **(A)** Effect of temperature on IOS production. The conditions of 15 h enzymatic hydrolysis were as follow: 30 g/L inulin, 10 U endo-inulinase per gram of inulin and pH 4.6. **(B)** Effect of inulin concentration of IOS production. The conditions of 15 h enzymatic hydrolysis were as follow: 10 U endo-inulinase per gram of inulin, pH 4.6 and 50°C. **(C)** Effect of enzyme loading on IOS production. The conditions of 15 h enzymatic hydrolysis were as follow: 40 g/L inulin, pH 4.6 and 50°C. **(D)** Time course of IOS production by different enzyme loading of endo-inulinase. The hydrolytic conditions were as follow: 40 g/L inulin, pH 4.6 and 50°C.

To further demonstrate effective IOS production using the enzyme preparation which was dominant in endo-inulinase activity (P70), enzymatic hydrolysis conditions (temperature, inulin concentration, enzyme loading, and reaction time) were investigated and optimized. As shown in Figure 5A, temperatures demonstrate notable influences on both IOS concentrations and yields, and the optimum temperature for inulin hydrolysis was 50°C. At this temperature, a maximum IOS yield of 69.7% and maximum IOS concentration of 20.9 g/L was achieved. Next, the effect of the inulin concentration was investigated in the range of 30–70 g/L. Figure 5B shows that an IOS yield of 68% was obtained at 40 g/L inulin. When the inulin concentration was higher than 40 g/L, the IOS yield decreased gradually. However, the IOS yields were in the range of 60% to 70%. This result suggests that the inulin concentration has a little effect on IOS yield.

Concerning optimal enzyme loading (Figure 5C), IOS yields were unsurprisingly larger (80%) when the enzyme loading was greater than 20 U/(g inulin). It can also be seen that the yield benefits to increasing enzyme loading beyond 20 U/(g inulin) were marginal at best, eliminating any expensive need to dose high levels of enzyme in a real process. Finally, the time course of inulin hydrolysis at different enzyme loadings is shown in Figure 5D. For the enzyme loading of 10 U/(g inulin), the maximum IOS concentration (25.3 g/L) was observed at 11 h with an IOS yield of 63.2%. Comparably, the maximum IOS concentrations (over 32 g/L) were observed at 9 h for all of the enzyme loadings between 20 U/(g inulin) and 50 U/(g inulin). This finding indicates that increasing endo-inulinase loading notably enhanced IOS concentrations and shortened required hydrolysis time at constant initial inulin concentrations. It should be pointed out that a higher IOS concentration (38.2 g/L) was observed at 9 h, representing the IOS yield of 95.4% at 40 U/(g inulin). Interestingly, further increasing hydrolytic time resulted in a slight decrease in IOS concentration. This result suggested that the endo-inulinase fraction did bear a slight level of exo-inulinase activity that could not be eliminated despite the fractionation protocol utilized. To minimize this occurrence, the optimal hydrolytic time was identified as 9 h.

### Distribution of Hydrolytic Products

**FIGURE 6:**
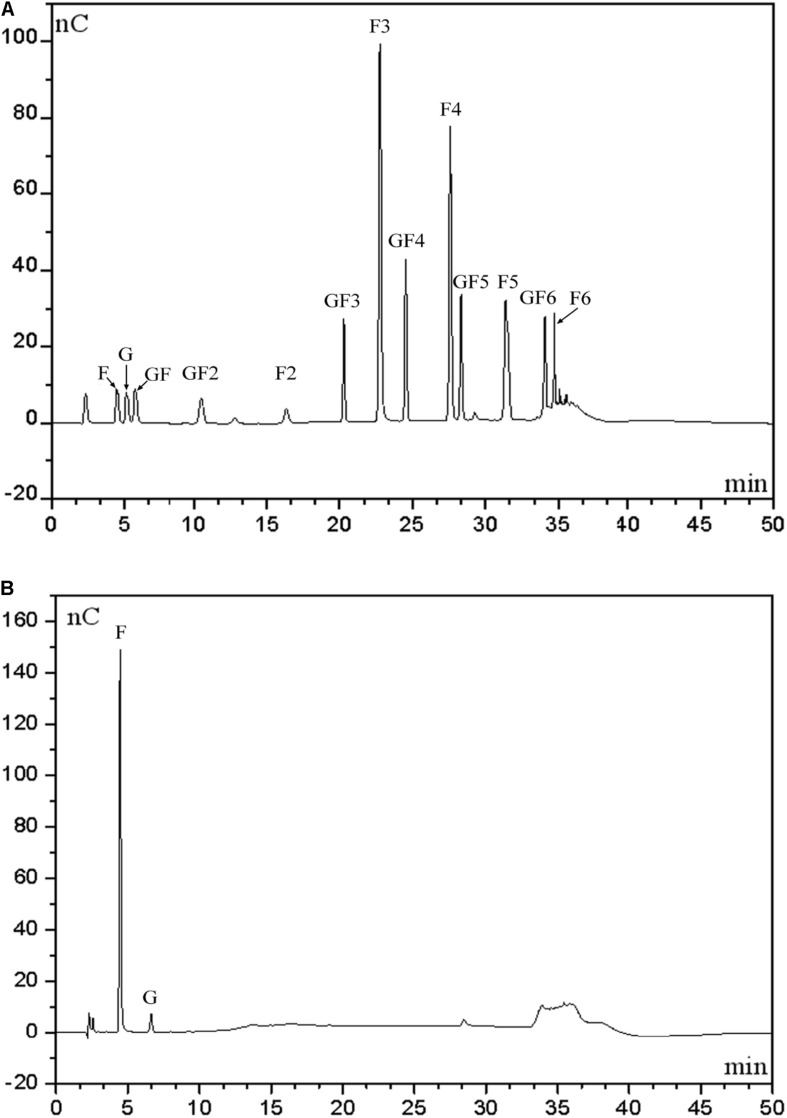
High-performance anion exchange chromatography of inulin hydrolysis products using fractionated endo-inulinase **(A)** and natural inulinase. **(B)** The hydrolysis conditions were: 40 g/L inulin, pH 4.6, enzyme loading 40 U/(g inulin) and 50°C. Abbreviations: glucose, G; sucrose, GF; 1-kestose, GF2; 1-nystose, GF3; 1-F-1-β-D-fructofuranosyl nystose, GF4; 1-F-(1-β-D-fructofuranosyl) -2-nystose, GF5; 1-F-(1-β-D-fructofuranosyl)-3-nystose, GF6; fructose, F; inulobiose, F2; inulotriose, F3; inulotetraose, F4; inulopentaose, F5; inulohexaose, F6.

To determine distribution of hydrolytic products produced by our endo-inulinase, a separate hydrolysis experiment using fresh enzyme and inulin was carried out with 40 g/L inulin, enzyme loading of 40 U/(g inulin), at pH 4.6, and 50°C for a total of 9 h. For comparison, a similar experiment was also performed using the natural inulinase. The distributions of reaction products in each hydrolyzate are shown in Figure 6. Beginning with Figure 6A, the major products produced using the endo-inulinase were oligosaccharides ranging from DP 2 to DP 7, which represented more than 94% of the hydrolyzate. Within this DP range, 85% of hydrolyzate components were between DP 4 and DP 7. The overall distribution of hydrolysis products was as follows: GF3 4.7%, inulotriose (F3) 26.4, GF4 9%, inulotetraose (F4) 19.3, GF5 7, inulopentaose (F5) 13.1, and GF6 5.5%. In contrast, fructose and glucose were the main products from inulin hydrolysis using natural inulinase (Figure 6B). Both of them contributed over 87% of hydrolyzate components, and little oligosaccharides were found in the hydrolyzate. This result means natural inulinase hydrolyzed inulin into monosaccharides within a 9-h enzymatic hydrolysis.

It has been reported that endo-inulinases purified from different microorganisms can produce IOS from inulin at yields ranging from 70 to 92% (Singh et al., 2016). In these cases, an endo-inulinase of *Aspergillus ficuum* was purified by column chromatography, and then used to hydrolyze inulin (Jin et al., 2005). In said study, the IOS products mainly consisted of DP 3 and DP 4 oligosaccharides, and the yield of 86%, major products of DP 5 and DP 6, was obtained by the endo-inulinase from *Xanthomonas* sp., which was purified by a DEAE-Sepharose CL 6B chromatography (Park et al., 1999); A dual endo-inulinase system from *Xanthomonas* sp. and *Pseudomonas* sp. was developed for IOS production with a enzyme dosage of 460 U/(g substrate). The IOS yield and DP value of major hydrolytic products after 110-h hydrolysis were 92% and equal or greater than 5, respectively (Cho et al., 2001). Some other attempts were carried out to yield IOS from inulin by recombinant endo-inulinase. Xu et al. (2016) reported DP values ranged from DP 3 to DP 6 produced by the recombinant endo-inulinase from *A. niger* DSM 2466 with a IOS yield of 91.3%. He et al. (2014) reported that the recombinant endo-inulinase from *A. niger* CICIM F0620 was applied for IOS production with a IOS yield of 91% and hydrolytic products ranged from DP 2 to DP 5 (He et al., 2014).

A higher IOS yield (∼94%) and a reasonable distribution of hydrolyzate ranging from DP 2 to DP 7 were observed in this study. These results proved that separation of endo-inulinase and exo-inulinase activities by the inulin-mediated ethanol gradient precipitation could be an effective alternative method to those column chromatographic techniques, and achieve a higher IOS yield compared to those by recombinant endo-inulinase.

## Conclusion

Inulinases produced by natural *A. niger* contained both endo- and exo-inulinase actvities. For IOS production from inulin, it is desirable to use only endo-inulinase as exo-inulinase would produce mainly the monosacchairdes fructose and glucose from inulin. In the present study, we developed a simple fractionation method using ethanol precipitation to separate endo- and exo-inulinases present in inulinases. the enriched endo-inulinase produced an IOS product containing DP 2 to DP 7 at a high yield of ∼94%.

## Data Availability Statement

The original contributions presented in the study are included in the article/Supplementary Material, further inquiries can be directed to the corresponding author/s.

## Author Contributions

WW and QZ: investigation and formal analysis. XL: supervision. XL and WW: writing – original draft. S-TY and XL: writing – review and editing. All authors contributed to the article and approved the submitted version.

## Conflict of Interest

The authors declare that the research was conducted in the absence of any commercial or financial relationships that could be construed as a potential conflict of interest.
