# Supplementary material for: A Novel Inulin-Mediated Ethanol Precipitation Method for Separating Endo-Inulinase From Inulinases for Inulooligosaccharides Production From Inulin
Source: Front Bioeng Biotechnol. 2021 Apr 29;9:679720. doi: 10.3389/fbioe.2021.679720 (PMC8116588; doi:10.3389/fbioe.2021.679720)
Supplement: Supplementary file 1 [file Table_1.DOCX]

Supplementary Material

# Supplementary Figures


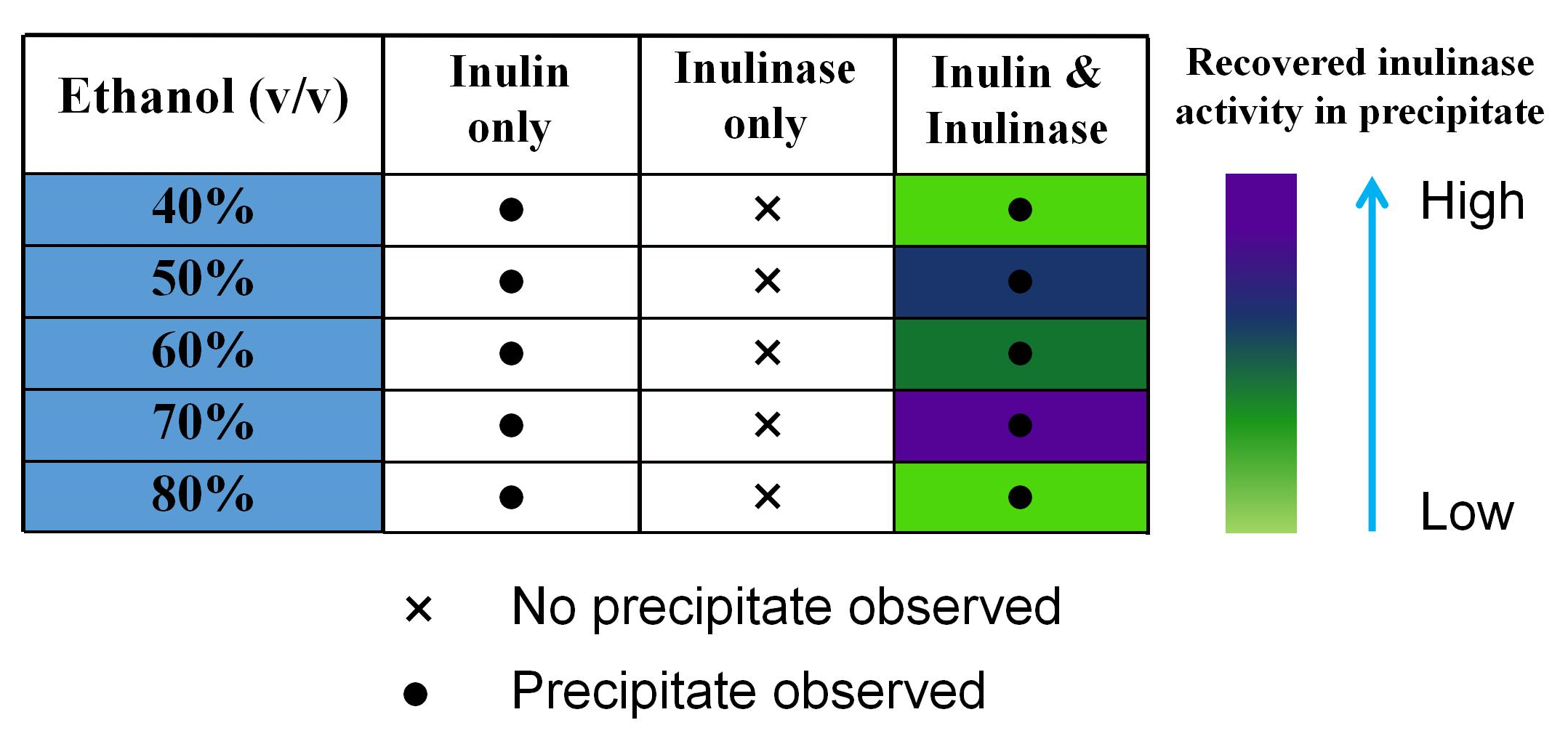


**Supplementary Figure S1.** Ethanol gradient precipitation of native inulinase both in the presence and absence of inulin.
